# Repairing and Analgesic Effects of Umbilical Cord Mesenchymal Stem Cell Transplantation in Mice with Spinal Cord Injury

**DOI:** 10.1155/2020/7650354

**Published:** 2020-04-04

**Authors:** Ling-ling Wu, Xiao-ming Pan, Hao-hao Chen, Xiao-yan Fu, Jinzhan Jiang, Ming-xing Ding

**Affiliations:** ^1^Medical Molecular Biology Laboratory, School of Medicine, Jinhua Polytechnic, Jinhua 321007, China; ^2^Jinhua Center of Laboratory Animals, Jinhua Municipal Food and Drug Inspection Institute, Jinhua, 321000 Zhejiang Province, China

## Abstract

Transplantation of human umbilical cord mesenchymal stem cells (hUC-MSCs) into spinal cord injury (SCI) may alleviate neuropathic pain and promote functional recovery. The underlying mechanism likely involves activation of glial cells and regulation of inflammatory factors but requires further validation. SCI was induced in 16 ICR mice using an SCI compression model, followed by injection of lentiviral vector-mediated green fluorescent protein- (GFP-) labeled hUC-MSCs 1 week later. Behavioral tests, histological evaluation, and inflammatory factor detection were performed in the treatment (SCI+hUC-MSCs) and model (SCI) groups. Histological evaluation revealed GFP expression in the spinal cord tissue of the treatment group, implying that the injected MSCs successfully migrated to the SCI. The Basso, Beattie, and Bresnahan (BBB) scores showed that motor function gradually recovered over time in both groups, but recovery speed was significantly higher in the treatment group than in the model group. The pain threshold in mice decreased after SCI but gradually increased over time owing to the self-repair function of the body. The corresponding pain threshold of the treatment group was significantly higher than that of the model group, indicating the therapeutic and analgesic effects of hUC-MSCs. Expression of IL-6 and TNF-*α* in the spinal cord tissue of the treated group decreased, whereas glial cell line-derived neurotrophic factor (GDNF) expression along with ED1 expression increased compared with those in the model group, suggesting that SCI activated ED1 inflammatory macrophages/microglia, which were subsequently reduced by hUC-MSC transplantation. hUC-MSCs are speculated to enhance the repair of the injured spinal cord tissue and exert an analgesic effect by reducing the secretion of inflammatory factors IL-6 and TNF-*α* and upregulating the expression of GDNF.

## 1. Introduction

After spinal cord injury (SCI), 53–80% of patients experience long-term moderate-to-severe pain [1, 2]. Such neuropathic pain occurs mostly in the area underneath the actual plane of SCI, where the pain sensation has disappeared. It is a stubborn complication post-SCI that seriously affects the patients' quality of life [3]. Since the mechanism of its occurrence remains unclear, traditional drugs and surgical treatment often fail to produce significant impact [4], and as a result, clinical treatment becomes very difficult.

Studies in recent years have shown remarkable effects of stem cell transplantation on the functional recovery from induced SCI, confirming that under special conditions, stem cells can proliferate and differentiate into nerve cells, such as mature neurons or glial cells [5, 6], thereby enhancing possibilities of recovery. However, relatively less attention has been paid to the effects on pain [1, 5]. Stem cells have good therapeutic potential in the treatment of SCI. Bone marrow, adipose tissue, placenta, amniotic fluid, and umbilical cord are good sources of mesenchymal stem cells. Human umbilical cord mesenchymal stem cells (hUC-MSCs) have higher amplification capacity, stronger proliferation ability, and lower risk of bacterial/viral infection. In addition, hUC-MSCs induce immune response when injected into injured tissues [7, 8]. Therefore, promotion of their application would be of great value. However, more evidence is required to confirm whether they are beneficial to the treatment of neuropathic pain in patients with SCI [9]. Using ED1, a marker of activated microglia, Roh et al. [10] demonstrated that transplantation of human stem cells into the injured spinal cord can suppress mechanical allodynia, and this effect seems to be closely associated with the modulation of spinal cord microglia activity. However, Pourheydar et al. [11] found that transplanted mesenchymal cells could not relieve neuropathic pain and induce hyperalgesia.

This study first attempted to identify the isolated and purified hUC-MSCs using lentiviruses carrying green fluorescent protein (GFP). A mouse SCI model was established, and local injection of hUC-MSCs was performed to determine their effect on SCI-induced neuropathic pain and explore the mutual regulatory mechanisms between inflammatory/immune cells and microglia, so as to provide a basis for the use of stem cells in the treatment of neuropathic pain from SCI.

## 2. Materials and Methods

### 2.1. Materials

hUC-MSCs were provided by Jinhua Sidanmu Stem Cell Biotechnology Limited Company (Jinhua, China). A GFP-tagged lentiviral vector with a titer of 1 × 10^8^ TU/mL was produced by Hangzhou Hibio Biotech Co., Ltd. (Hangzhou, China). ICR mice were purchased from Shanghai SLAC Laboratory Animal Co., Ltd., with the Laboratory Animal Production License No. SCXK (Hu) 2012-0002 and Laboratory Animal Use License No. SYXK (Zhe) 2015-0008. DMEM, fetal bovine serum (FBS), trypsin, penicillin, and streptomycin were purchased from Gibco (Waltham, MA, USA). Fluorescein-conjugated antibodies against CD34, CD45, CD19, CD14, CD105, CD90, and CD73 were purchased from BD Biosciences (Franklin Lakes, NJ, USA). ED1 was purchased from Serotec Ltd. (Oxford, UK; MCA341GA). The IL-6, TNF-*α*, and glial cell line-derived neurotrophic factor (GDNF) ELISA kits were purchased from Hangzhou MultiSciences (Lianke) Biotech Co., Ltd. (Hangzhou, China; EK2016, EK2821, and EK0935). The main instruments included a CO_2_ incubator and microplate reader (Thermo Fisher Scientific, Waltham, MA), inverted fluorescence electron microscope (Leica, Wetzlar, Germany; DMI3000B), and laser confocal microscope (Olympus Corporation, Tokyo, Japan; IX83-FV3000).

### 2.2. Isolation, Culture, and Identification of hUC-MSCs

Umbilical cord tissue was obtained, washed with PBS thrice, and cut with ophthalmic scissors; the tissue was transferred to a cell separation tube, and 2 mL of basal medium (DMEM-F12+10%, FBS+1% penicillin, and streptomycin) was added and stirred thrice (for 45 s each time). The sample was then transferred to a 50 mL centrifuge tube to which an appropriate amount of PBS was added. The tissue was centrifuged at 300 × g for 15 min, the supernatant discarded, and residue washed thrice with PBS. The sample was added to the culture medium and cultured in an incubator at 37°С with 5% CO_2_. When the cell confluence reached ≥80%, they were collected and passaged until the third generation, after which they were collected for flow cytometry. Expression of cell surface molecules CD19, CD14, CD34, CD45, CD73, CD90, and CD105 was recorded.

### 2.3. Transfection of hUC-MSCs with Lentivirus Carrying GFP

After the identified cells were passaged and amplified, the cells were suspended to a density of 5 × 10^5^ cells/mL and inoculated into a 6-well plate; the medium was changed after 4 h. Part of the medium was discarded, and a virus with a multiplicity of infection (MOI) of 10 along with 8 ng/mL of polybrene (Sigma-Aldrich, St. Louis, MO, USA) was added. After 6 h culture, the medium was changed, and the culture continued for 72 h before collecting cells for subsequent experiments.

### 2.4. Establishment of SCI Model

Establishment of the contusive SCI mouse model was performed according to the methods proposed by Tu et al. with slight modification [12]. Sixteen ICR mice (15-week-old, male, 37–45 g) were divided into the model group and the treatment group (8 per group) and acclimatized for 1 week. The mice were anesthetized by intraperitoneal injection with pentobarbital sodium (60 mg/kg), and laminectomy was performed to expose the spinal cord at vertebrae T9–T10. The M-III Spinal Impactor (W.M. Keck Center for Collaborative Neuroscience) was used to induce contusive SCI by applying 90 kilodynes of force to the exposed spinal cord. Gentamicin (8 mg/kg) was intramuscularly injected daily for 10 days after inducing SCI to prevent postoperative infection, and artificial bladder compression was performed twice daily to express urine until spontaneous bladder activity was resumed to prevent urinary retention. After 1 week of treatment, the Basso, Beattie, and Bresnahan (BBB) scores were used to evaluate mobility of the posterior limbs of the mice; a BBB score of 0 indicated successful establishment of the model.

All animals were maintained under conventional housing conditions, with food and water available ad libitum in a temperature-controlled room with a 12 h light/dark cycle. The mice were fasted for 12 h before the experiments. All protocols and surgeries were approved by the Medical Ethics Committee of Jinhua Polytechnic (Jinhua, China) and were performed in accordance with the animal research regulations of the National Natural Science Foundation of China and the Guide for the Care and Use of Laboratory Animals issued by the National Institutes of Health.

### 2.5. Transplantation of hUC-MSCs and Grouping

After successful establishment of the SCI model, the mice were divided into two groups: the model group and the treatment group (8 mice/group). The model group was injected with 3 *μ*L of serum-free medium (without cells) at a speed of 1 *μ*L/min at the center of the injured spinal cord; as previously described [13], the treatment group was injected with 3 *μ*L of growth factor-free medium (containing 1.0 × 10^5^/*μ*L of hUC-MSCs) using a 29 G needle with a 10 *μ*L microinjection pump (Stoelting America, 51639) at a speed of 1 *μ*L/min at the center of the injured spinal cord. After infusion, the needle was left in place for 1 min to enable the solution to diffuse into the tissue. This injection was performed 1 week after inducing SCI, and the needle was retained for 5 min after the injection to reduce cell leakage. An immunosuppressive agent, cyclosporine (10 mg/kg), was injected daily after cell transplantation. After inducing SCI, 16 mice were maintained for 3 weeks, and based on the BBB scoring results, three were taken from each group during this time for immunofluorescence assay and ELISA. The remaining 10 mice were reserved for behavioral testing. At 9 weeks after inducing SCI, all injured spinal cord centers were paraffin embedded.

### 2.6. BBB Motor Function Scoring of Both Posterior Limbs in Mice [14]

Behavioral test data were collected from a total of 16 mice 2 weeks after injection of hUC-MSCs (i.e., 3 weeks after inducing SCI). The spinal cord tissue from three mice in each group (6 mice in total) exhibiting significant changes was selected for pathological examination. BBB scoring was performed for the remaining five mice per group (10 mice in total) every alternate week (i.e., 5, 7, and 9 weeks after inducing SCI). The scores ranged from 0 to 21 points, with 0 points indicating complete loss of motor function in the posterior limbs and 21 points indicating normal motor function of posterior limbs.

### 2.7. Mechanical Allodynia Tests

The test was performed following the method reported earlier, with slight modification [10]. Mice were placed on a metal grid shelf to allow them to adapt to the environment. von Frey hairs (Ugo Basile SRL, Gemonio VA, Italy) were used to stimulate the skin surface of posterior soles, vertically from the bottom of the soles. The pressure (g) causing paw withdrawal was recorded, with four measurements at 2 min intervals. The median value was obtained as the pain threshold (g), after excluding the maximum and minimum values.

### 2.8. GFP Immunofluorescence Assay

The spinal cord tissue was fixed by formaldehyde perfusion, externally fixed, and dehydrated with sucrose, and 15 *μ*m thick frozen sections were prepared on a cryostat microtome. Triton X-100 (1%) was used to permeabilize cell membranes at room temperature for 15 min. The sections were then washed with water for 5 min, treated with 3% hydrogen peroxide for 10 min, washed with 0.01 M PBS for 5 min, and blocked in 5% BSA for 1.5 h, after which the blocking solution was dried. DAPI was used to stain the nuclei, and photos were taken under a fluorescence microscope.

### 2.9. Laser Confocal Detection of ED1 Expression

The sections (described above) were incubated overnight with a primary antibody (monoclonal against ED1 clone), at a dilution of 1 : 200, in a freezer at 4°C. They were then incubated with a secondary antibody (Cy3-labeled goat anti-rabbit IgG (H+L) antibody), at a dilution of 1 : 500, at 4°C for 2 h and protected from light during the entire process. The sections were washed thrice with 0.01 M PBS for 5 min each time. DAPI was used to stain the nuclei, and photos were taken under a laser confocal microscope.

### 2.10. Western Blot Analysis

In brief, samples (cells or spinal cord segments at L1–L6) were collected and washed with ice-cold phosphate-buffered saline before being lysed in a radioimmunoprecipitation assay lysis buffer. Then, whole sample lysates were separated by SDS-PAGE and electrophoretically transferred onto a polyacrylamide gel. The membranes were blocked with 5% bovine serum albumin for 1 h at room temperature, probed with antibodies (Inos, ab15323, 1 : 500, Abcam; 5-HT3A, 10043-1-AP, 1 : 500, Proteintech) overnight at 4°C with the primary antibodies, and then incubated with secondary antibodies (Goat Anti-Rabbit IgG Antibody, BL003A, biosharp). Western blot analyses were replicated in 4 independent experiments.

### 2.11. ELISA of IL-6, TNF-*α*, and GDNF

An appropriate amount of tissue block was taken, washed in precooled PBS, and transferred to a glass homogenizer placed in ice, after removing the blood. Five to ten milliliters of precooled PBS was added for thorough grinding, the entire process being carried out on ice. The prepared tissue homogenate was centrifuged at 5000 rpm for 5 min, and the supernatant was preserved for the test. Levels of IL-6, TNF-*α*, and GDNF in the spinal cord tissue of each group of mice were detected using specific ELISA kits, in strict accordance with the manufacturer's instructions.

### 2.12. Histologic Analysis of Injury Area

Histologic analysis was performed on the SCI longitudinal sections to compare the size of the site of injury between the treated and model groups. Sections from model and treated mice with hMSCs were stained with hematoxylin and eosin (H.E. staining).

### 2.13. Statistical Analyses

Statistical analysis was performed using SPSS 22.0 software (IBM Corp., Armonk, NY, USA). Data are expressed as mean ± standard deviation. Between groups comparison was made using unpaired Student's *t*-test. Within group comparison was made using repeated measures of ANOVA followed by Tukey's HSD test. *P* < 0.05 was considered to imply statistically significant differences.

## 3. Results

### 3.1. Identification of hUC-MSCs by Flow Cytometry

**Figure 1:**
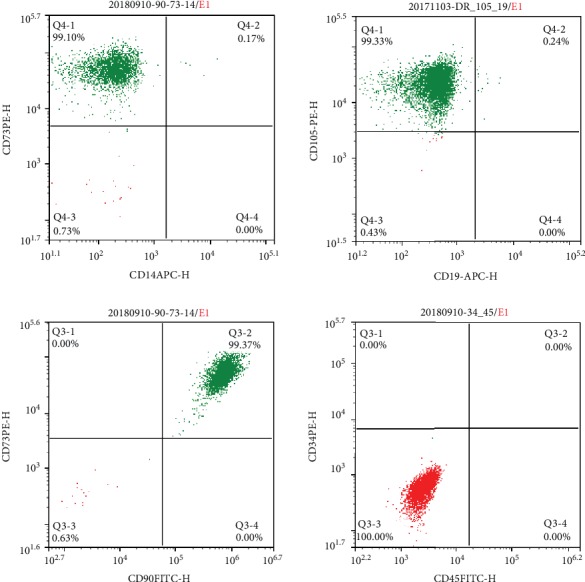
Identification of mesenchymal stem cells by flow cytometry. (a) CD14 (APC) was negative and CD73 (PE) was positive; (b) CD19 (APC) was negative and CD105 (PE) was positive; (c) CD34 (PE) and CD45 (FITC) were both negative; (d) CD90 (FITC) was positive.

Flow cytometry results indicated the cell surface marker CD14 (APC) to be negative and CD73 (PE) to be positive (Figure 1(a)). Similarly, CD19 (APC) was negative and CD105 (PE) positive, CD90 (FITC) was positive, and both CD34 (PE) and CD45 (FITC) were negative (Figure 1). Based on this, the cells were confirmed as hUC-MSCs.

### 3.2. Establishment of hUC-MSCs with High GFP Expression

**Figure 2:**
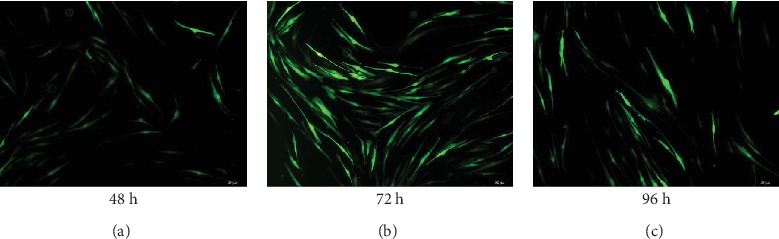
GFP expression in umbilical cord mesenchymal stem cells at various time points. hUC-MSCs were infected with GFP lentiviruses and observed for 48 h, 72 h, and 96 h. GFP green fluorescence was expressed in the cells at (a) 48 h, (b) 72 h, and (c) 96 h, expression being the highest in cells infected for 72 h. Scale bars: 50 *μ*m.

As shown in Figure 2, when the infection time was 48 h, 72 h, and 96 h, all three groups of cells had GFP expression (Figure 2), indicating that GFP had been successfully inserted into hUC-MSCs. Comparing fluorescence intensities at the three different time points, green GFP fluorescence was obviously the highest in cells infected for 72 h. Therefore, the optimal infection time for GFP lentivirus-infected hUC-MSCs was 72 h, which was adopted for the subsequent amplification of cells.

### 3.3. BBB Scoring for Motor Function of Posterior Limbs in Mice

**Figure 3:**
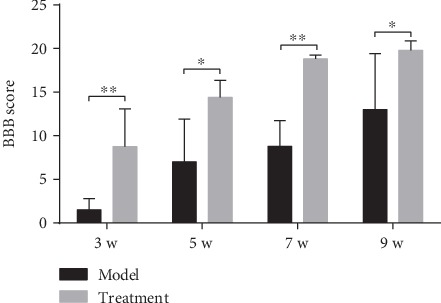
BBB scores in each group of mice at different time points. Two weeks after hUC-MSC transplantation (3 weeks after SCI), each group of mice underwent BBB (Basso, Beattie, and Bresnahan) test every alternate week (5, 7, and 9 weeks after SCI). Compared to the model group, the BBB scores of the treatment group were significantly increased at 3, 5, 7, and 9 weeks (*P* < 0.05 or *P* < 0.01; *n* = 8/group). Data are expressed as the mean ± SD, and repeated measures analysis of variance (ANOVA) and Tukey's one-way analysis of variance for post hoc testing were used for analyses. ^∗∗^*P* < 0.01; ^∗^*P* < 0.05. hUC-MSCs: human umbilical cord mesenchymal stem cells; BBB: Basso, Beattie, and Bresnahan.

BBB scores of the two groups of mice at various time points showed that motor function of the model and treatment groups gradually recovered over time (*P* < 0.01). The recovery rate of motor function in the treatment group was significantly higher than that in the model group. BBB scores of the treatment group at 3, 5, 7, and 9 weeks were significantly higher than those of the model group (*P* < 0.05 or *P* < 0.01) (Figure 3).

### 3.4. Mechanical Allodynia Test

**Figure 4:**
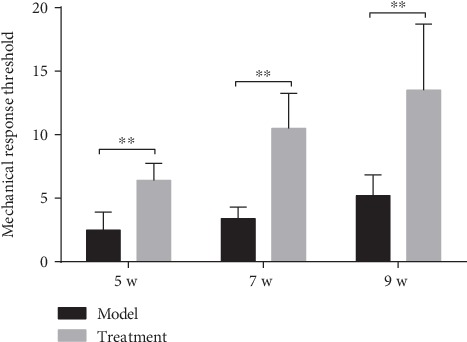
Results of pain threshold measurement in each group of mice at each time point. Four weeks after hUC-MSC transplantation (5 weeks after SCI), mice in each group were tested every two weeks (5, 7, and 9 weeks after SCI). The von Frey wire was used to vertically stimulate the skin surface under the foot of the hind paw, and the pressure (g) at which paw withdrawal occurred was recorded. The median value was measured four times at 2 min intervals to get the pain threshold (g). For the same time point, the pain threshold of the hUC-MSC treatment group was significantly higher than that of the model group (*P* < 0.01; *n* = 8/group). Data are expressed as the mean ± SD, and repeated measures analysis of variance (ANOVA) and Tukey's one-way analysis of variance for post hoc testing were used. ^∗∗^*P* < 0.01.

At each time point (5, 7, and 9 weeks) after SCI, the pain threshold of the two groups of mice was lower. With the passage of time, the pain threshold gradually increased owing to the self-repair function of the body (*P* < 0.01). For the same time point, the pain threshold of the hUC-MSC treatment group was significantly higher than that of the model group (*P* < 0.01) (Figure 4), indicating therapeutic and analgesic effects of hUC-MSCs on the injured spinal cord.

### 3.5. GFP and GFAP Immunofluorescence Assay

**Figure 5:**
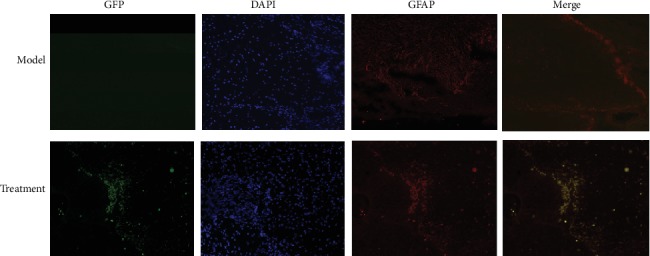
GFP and GFAP expression in the spinal cord tissue of each group. In the frozen sections of the spinal cord tissue, DAPI was used to stain the nucleus, and the expression of GFP and GFAP in the tissue of the model and treatment groups was detected by fluorescence microscopy. While there was no GFP expression in the spinal cord of the model group, a small amount of GFP/GFAP immunofluorescence double-positive cells was shown in the treatment group. DAPI: 4,6-diamino-2-phenylindole; GFP: green fluorescent protein; GFAP: glial fibrillary acidic protein. Scale bars: 100 *μ*m.

While GFP was not expressed in the spinal cord tissue of the model group, a small amount of GFP/GFAP immunofluorescence double-positive cells was shown in the treatment group (Figure 5). This observation indicated that the hUC-MSCs carrying GFP were successfully injected and had proliferated in the injured spinal cord and could be induced to differentiate into glial cells, thereby promoting its repair.

### 3.6. Laser Confocal Detection of ED1 Expression

**Figure 6:**
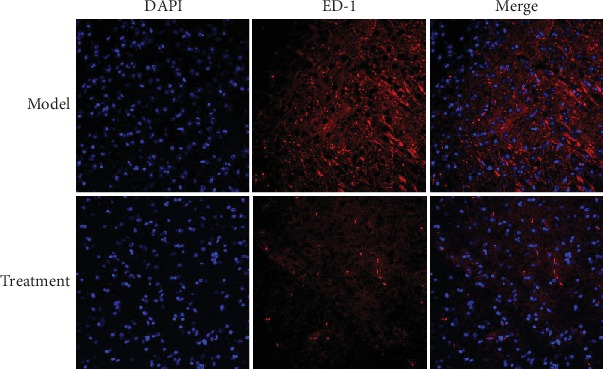
ED1 fluorescence expression in the spinal cord tissue of each group. Frozen sections of the spinal cord tissue were stained with primary anti-rabbit source ED1 (1 : 200) and secondary antibody Cy3-labeled goat anti-rabbit IgG (H+L) (1 : 500); the nucleus was stained with DAPI, and ED1 fluorescence in the spinal cord tissue was higher in the model group than in the treatment group. Scale bars: 50 *μ*m.

High ED1 fluorescence was observed in the spinal cord tissue of the model group, whereas that in the treatment group was reduced (Figure 6).

### 3.7. Western Blot for 5-HT3A and iNOS Expression in the Spinal Cord Tissues of Mice

**Figure 7:**
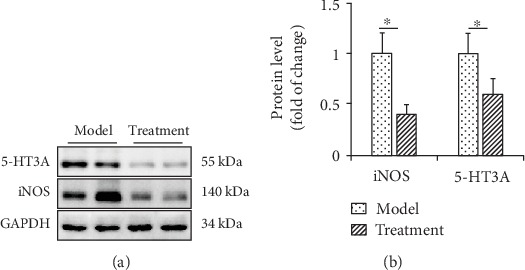
Effect of hUC-MSC transplantation on the protein expression of 5-HT3A and iNOS in the spinal cord of mice. (a) Western blot was performed to evaluate the levels of 5-HT3A and iNOS in protein extracted from the L1–L6 segment of the spinal cord in each group. GAPDH was used as an internal control. (b) Statistical analysis of data obtained from western blotting experiments was performed using unpaired Student's *t*-test. Data are presented as the mean ± standard error mean. *n* = 8 each. ^∗^*P* < 0.05, vs. model.

The expression of GAPDH was more consistent; one week after hMSC transplantation, protein expression levels of 5-HT3A and iNOS were lower in the spinal cord tissues of the hUC-MSC group than in those of the model group (*P* < 0.05) (Figure 7).

### 3.8. ELISA for IL-6, TNF-*α*, and GDNF Expression in the Spinal Cord Tissues of Mice

**Figure 8:**
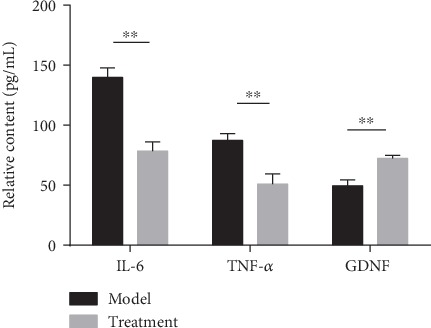
Expression of IL-6, TNF-*α*, and GDNF in the spinal cord tissue of each group. Two weeks after hUC-MSC transplantation, the levels of IL-6, TNF-*α*, and GDNF in the spinal cord of each group were detected by ELISA. Compared to the model group, the expression of IL-6 and TNF-*α* in hUC-MSCs was significantly decreased while that of GDNF was significantly increased (all *P* < 0.01; *n* = 8/group). Data are expressed as the mean ± SD, and unpaired analysis by Student's *t*-test was done. ^∗∗^*P* < 0.01; IL-6: interleukin 6; TNF-*α*: tumor necrosis factor alpha; GDNF: glial cell-derived neurotrophic factor.

Figure 8 shows the expression of IL-6, TNF-*α*, and GDNF in the spinal cord tissue of each group. Expression of IL-6 and TNF-*α* was significantly decreased, while the expression of GDNF was significantly increased in the hUC-MSC treatment group, compared to the model group, after 2 weeks of treatment (all *P* < 0.01). GDNF might promote the repair of the injured spinal cord. We therefore speculated that hUC-MSCs may promote the repair of the injured spinal cord tissue by reducing the secretion of inflammatory factors IL-6 and TNF-*α* and increasing the expression of GDNF.

### 3.9. Histologic Analysis

**Figure 9:**
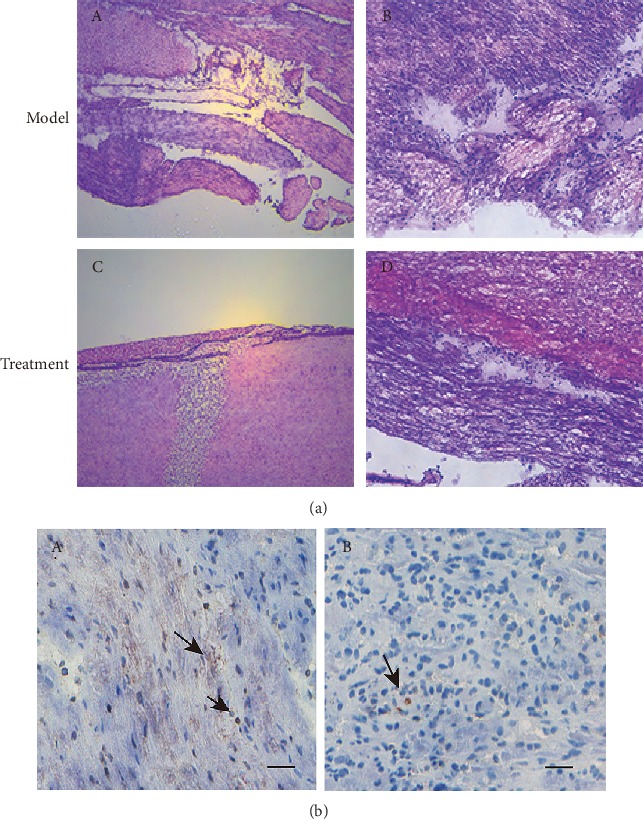
Histological comparison of the spinal cord tissue in each group. (a) The gray matter in the spinal cord of the (A, B) model group was unclear. The vacuolar necrosis was observed, the nerve fibers were relatively sparse and chaotic, and there were different degrees of inflammatory cell infiltration. In the (C, D) treatment group, the inflammatory cells infiltrated decreased, and the nerve fibers passed through the spinal cord injury area of mice. H.E. staining. Scale bars: 50 *μ*m. (b) Immunohistochemical staining for inflammatory cell identification of CD15 (mouse monoclonal anti-CD15, 1 : 350, Abcam) in sections of mouse spinal cord. Note that a more intense CD15-positive cell (arrowhead) response was shown in the (A) model group, but poor CD15-positive cell (arrowhead) response at or proximal to the zone of injury in the (B) treatment group. Scale bars: 100 *μ*m.

The spinal cord sections showed different degrees of inflammatory reaction in the model group and the treatment group (Figure 9). Treatment with hUC-MSCs was able to significantly reduce inflammatory cell infiltration.

## 4. Discussion

Whether transplantation of hUC-MSCs can alleviate neuropathic pain due to SCI and promote functional recovery is not yet clear. The mechanism may be related to the activation of glial cells and regulation of inflammatory factors after SCI; hUC-MSCs are also known to improve microcirculation and the microenvironment through therapeutic paracrine effects, although more evidence is required [6, 15]. In this study, an SCI compression model was used, and lentiviral vector-mediated GFP-labeled hUC-MSCs were transplanted into the injured spinal cord. MSCs were found to successfully migrate to the injured site, with certain analgesic and therapeutic effects on the damaged spinal cord. They also reduced the activation level of ED1 inflammatory macrophages/microglia in the injured spinal cord, inhibited the secretion of inflammatory factors IL-6 and TNF-*α*, and increased the expression of GDNF.

Cell transplantation is currently considered a preferred treatment for SCI. In fact, studies have shown strong effects of stem cell transplantation on functional recovery after inducing SCI. However, the fate of stem cells in vivo varies with the intrinsic properties of the cells and sites of transplantation [16]. Therefore, the best sources of stem cells and their transplantation pathways remain controversial in the treatment of SCI. Our current findings provide evidence that transplanted stem cells can migrate to the site of injury to reduce neuropathic pain. Furthermore, GFP and GFAP double staining showed good results, suggesting that it has the potential of glial differentiation. Recently, Yousefifard et al. [17] used a rat SCI model to confirm that both bone marrow MSC and UC-MSC transplantation alleviates symptoms of neuropathic pain and results in motor function recovery after SCI. Moreover, UC-MSCs are supported by more survival rate and electrophysiological data and have advantages over bone marrow MSCs [18]. Chen et al. [19] found that in a rat pain model induced by nerve ligation, continuous intrathecal injection of UC-MSC exosomes achieved excellent prevention and reversal of pain caused by nerve ligation. Therefore, this approach could be a new treatment for pain from nerve injury [20, 21]. Clinical studies have also shown that the intensity of neuropathic pain gradually improves after the administration of MSCs, supporting the notion that intrathecal injection of autologous MSCs into the spinal cord is beneficial to patients with SCI [13, 20]. In addition, hUC-MSCs induce immune response when injected into injured tissues [7]. When MSCs are used for coculture with other cells, or for stents, they can improve the outcome of SCI surgery, making it safer and more effective, and also reduce morbidity caused by the surgery [21, 22].

Our results also demonstrated that the injured spinal cord causes activation of ED1 inflammatory macrophages/microglia, whereas hUC-MSCs reduce their activation levels, downregulate the expression of IL-6 and TNF-*α*, and upregulate the expression of GDNF, which may promote the repair of the injured spinal cord tissue and produce an analgesic effect. Neuropathic pain after SCI is speculated to be associated with neuronal protection, inflammation and signaling pathways, and local microenvironmental changes, though more evidence is required. During the acute phase of SCI, damage to the blood-spinal cord barrier causes severe immunoinflammatory response, making the microenvironment unsuitable for the survival and differentiation of MSCs, whereas if SCI persists for too long, scar tissue may form, which is not conducive to the growth of axons. Therefore, the best timing for transplantation is within 1–2 weeks of SCI [23, 24]. During this period, the damage to transplanted cells by inflammatory factors can be relieved, and the impact of scar tissue on axon growth can be avoided. At present, the main methods of cell transplantation include transplantation by intravenous infusion and transplantation at the injured site. Although intravenous infusion of stem cell suspension is simple, practicable, and less risky, the number of cells predicted to reach the injured site is not always accurate. Injured-site transplantation, used here, can ensure direct delivery of stem cells into the spinal cord, with a clear effect. In fact, intrathecal administration of MSCs by lumbar puncture is very useful and feasible for MSC treatment of brain damage, such as in stroke or neurodegenerative diseases [25]. Chen et al. [19] found that intrathecal transplantation significantly improved spinal nerve ligation- (SNL-) induced mechanical allodynia and thermal hyperalgesia. The mechanism is related to the inhibition of neuroinflammation, including inhibition of activated astrocytes and microglia, as well as significant reduction of inflammatory cytokines IL-1*β* and IL-17A and upregulation of anti-inflammatory cytokine IL-10. GDNF is a confirmed nerve growth and survival factor. In SCI animal experiments, both subcutaneous adipose tissue- and bone marrow-derived MSCs have been confirmed to increase GDNF protein expression and enhance angiogenesis and functional recovery, demonstrating the therapeutic potential of MSCs for SCI [26, 27]. At the same time, transplantation of hUC-MSCs in SCI rats and mice can enhance the anti-inflammatory effect, antiastrocytic proliferation, antiapoptosis effect, and axonal retention by promoting polarization of M2 macrophages [13]. The most relevant neuropeptides for neuropathic pain are 5-HT and nitric oxide (NO). NOS is a key enzyme in the synthesis of NO, which can catalyze the production of citrulline by L-arginine (L-Arg) and release endogenous NO. The increase of NOS activity is closely related to the maintenance of the hyperalgesia state. The spinal cord contains a variety of subtypes of 5-HT and its receptors, which have different effects in the pathogenesis of neuropathic pain [28]. Meanwhile, our results show that after stem cell transplantation, iNOS and 5-HT3A expression of the spinal cord tissues is decreased, thereby exerting an analgesic effect. These results suggest that 5-HT3A is involved in the development of hyperalgesia by regulating the expression of NOS or has a harmonized role in the spinal cord. Thus, hUC-MSC transplantation has beneficial effects on functional recovery after SCI, promoting repair of the injured site and improving motor function [13, 29].

## 5. Conclusions

In summary, hUC-MSC implantation is effective for recovery of motor function in SCI, while targeted intrathecal hUC-MSCs can provide a new therapeutic strategy for neuropathic pain relief through effects on glial cells, proinflammatory cytokines, and anti-inflammatory cytokines.

## Data Availability

The data used to support the findings of this study are available from the corresponding author upon request.

## Abbreviations

BBB: Basso, Beattie, and Bresnahan.
SCI: Spinal cord injury.

## Additional Points

*Highlights*. hUC-MSC transplantation for treating spinal cord injury in rats was studied. Injected MSCs carrying GFP fluorophores proliferated in the injured spinal cords. Motor function recovered faster and pain threshold was higher in the transplanted group. hUC-MSC transplantation reduced the expression of genes induced by SCI. hUC-MSCs may enhance spinal cord repair and exert an analgesic effect.
